# Quantitative Phosphoproteomics Analysis of Nitric Oxide–Responsive Phosphoproteins in Cotton Leaf

**DOI:** 10.1371/journal.pone.0094261

**Published:** 2014-04-08

**Authors:** Shuli Fan, Yanyan Meng, Meizhen Song, Chaoyou Pang, Hengling Wei, Ji Liu, Xianjin Zhan, Jiayang Lan, Changhui Feng, Shengxi Zhang, Shuxun Yu

**Affiliations:** 1 State Key Laboratory of Cotton Biology, Institute of Cotton Research, Chinese Academy of Agricultural Sciences, Anyang, Henan Province, China; 2 Key Laboratory of Cotton Biology and Breeding in the Middle Reaches of the Changjing River, Institute of Economic Crop, Hubei Academy of Agricultural Science, Wuhan, Hubei Province, China; 3 College of Agronomy, Northwest A & F University, Yangling, Shaanxi Province, China; Universität Regensburg, Germany

## Abstract

Knowledge of phosphorylation events and their regulation is crucial to understanding the functional biology of plant proteins, but very little is currently known about nitric oxide–responsive phosphorylation in plants. Here, we report the first large-scale, quantitative phosphoproteome analysis of cotton (*Gossypium hirsutum*) treated with sodium nitroprusside (nitric oxide donor) by utilizing the isobaric tag for relative and absolute quantitation (iTRAQ) method. A total of 1315 unique phosphopeptides, spanning 1528 non-redundant phosphorylation sites, were detected from 1020 cotton phosphoproteins. Among them, 183 phosphopeptides corresponding to 167 phosphoproteins were found to be differentially phosphorylated in response to sodium nitroprusside. Several of the phosphorylation sites that we identified, including RQxS, DSxE, TxxxxSP and SPxT, have not, to our knowledge, been reported to be protein kinase sites in other species. The phosphoproteins identified are involved in a wide range of cellular processes, including signal transduction, RNA metabolism, intracellular transport and so on. This study reveals unique features of the cotton phosphoproteome and provides new insight into the biochemical pathways that are regulated by nitric oxide.

## Introduction

Nitric oxide (NO) is an important growth regulator that modulates diverse developmental processes in plants over the entire lifespan, including cell elongation and germination [1], root development [2], stomatal movement [3], pollen tube growth [4], and plant senescence [5]. NO is also involved in plant responses to abiotic stresses such as heavy metal exposure [6], low temperature [7], salt [8], and drought [9]. Additionally, NO has been demonstrated to play many roles in biotic interactions, such as fighting pathogen infections [10], [11], deterring herbivore feeding [12] and building symbiotic interactions [13].

During the last decade, several medium- and large-scale transcriptomic analyses have identified hundreds of putative NO-regulated genes [14]–[19]. Moreover, eight families of transcription factor–binding sites have been identified in the promoter of NO-regulated genes [19]. Recent research has mainly focused on identifying NO target proteins, attempting to reveal NO involvement in plant cell biology. As such, more than 200 NO-responsive proteins have been identified in plants [20], [21]. Unlike the genome, however, protein abundance, structure, stability, subcellular localization, and interactions with other biological macromolecules are in continuous flux and these changes are regulated by post-translational modifications such as phosphorylation, acetylation, glycosylation, and methylation.

Phosphorylation is the most common and the most important dynamic adjustment mechanism and is involved in nearly all cellular and extracellular processes including defense responses, signal transduction, cytoskeleton regulation, and apoptosis [22], [23]. Given the importance of phosphorylation, there has been much interest in identifying new phosphorylated proteins and phosphorylation sites and in exploring the functional role of these phosphoproteins. Early studies looking at the effect of abscisic acid (ABA) on protein phosphorylation of rice (*Oryza sativa*) and *Arabidopsis thaliana* have identified 6 and 50 proteins, respectively, for which phosphorylation was found to be regulated by ABA [24], [25]. Furthermore, examination of changes in the phosphoproteome of rape seeds during the filling stage identified 70 phosphoproteins and 16 non-redundant phosphoproteins, which were verified by mapping the phosphorylation sites [26]. Analysis of vacuolar and cell membranes of rice bud and root revealed 230 membrane and membrane-associated proteins, 20% of which are phosphorylated [27]. In addition, soybean (*Glycine max*) root hairs contain 1625 unique phosphopeptides, including 1659 non-redundant phosphorylation sites, which originate from 1126 phosphoproteins [28]. A recent study identified multiple components of ABA-responsive protein phosphorylation network by integrating genetics with phosphoproteomics [29]. Furthermore, Wang et al. have shown the role of the SnRK2 protein kinases in the ABA signaling pathway by using quantitative phosphoproteomics [30]. To date, only a few plant species have been annotated with protein phosphorylation data and these do not include the economically important plant, cotton (*Gossypium hirsutum*) [31]. We previously examined the proteome of cotton leaves in response to NO treatment [21]. To follow-up this research, we performed quantitative time-course measurements of the phosphoproteome of cotton leaves treated with sodium nitroprusside (SNP), a NO donor. This is the first study to show that NO exposure in leaf tissue alters the phosphorylation states of multiple proteins found in cotton. This information should accelerate research on NO metabolic regulation and will lay a novel, theoretical foundation for further related studies in cotton.

## Materials and Methods

### Plant materials

Seeds of *G. hirsutum* ecotype CCRI10 were cultured in a mix of sand and nutritional soil in a culture room under white fluorescent light (14 h light/10 h dark) with day/night temperatures of 30/22°C. Previously we evaluated the effects of nitric oxide on cotton seeds treated with 0, 0.05, 0.1 or 0.5 mM SNP for 24 h. The results showed that the effect of treatment with 0.05 mM SNP on plant growth was not severe, 0.5 mM SNP was lethal, and 0.1 mM SNP was significant but not toxic (Figure S1). Additionaly, we also found that the effect of treatment at 0.1 mM SNP for 6 h on cotton seedlings effected protein levels significantly [21]. Based on these results, proteins were extracted from the leaves treated with 0.1 mM SNP. Plants that were 30 days old after sowing were irrigated with 0.1 mM SNP (Sigma, USA) in distilled water for 0 h (control), 1 h, 3 h and 6 h, respectively during the light period. The experiment was performed in triplicate with 30 plants in each group. Fresh, fully expanded leaves were harvested after treatment and immediately frozen in liquid nitrogen and stored at −80°C.

### Protein extraction

Leaf samples were ground in liquid nitrogen and lysed in buffer containing 4% SDS, 100 mM Tris-HCl, and 1 mM dithiothreitol (pH 7.6) at room temperature and briefly sonicated to reduce the viscosity of the lysate. The ratio of buffer to tissue was 5∶1 (v/v). After 3 min incubation in boiling water, the suspensions were ultrasonicated (80 w, 10 s ultrasonic at a time, every 15 s, and 10 times) then incubated at 100°C for 3 min. The crude extract was clarified by centrifugation at 13,000×*g* at 25°C for 10 min [32]. The sample protein content was determined using the BCA Protein Assay Reagent (Promega, USA). The supernatants were stored at −80°C until use.

### Protein digestion

Protein digestion was processed by using the method of filter-aided sample preparation (FASP) [32]. The protocol was as follows: For each sample, 300 μg of protein was diluted with 200 μL UA buffer (8 M urea with 0.1 M dithiothreitol in 0.15 M Tris-HCl pH 8.0) and centrifuged at 14,000×*g* at 20°C for 40 min. Another 200 μL of UA buffer was added and the samples were centrifuged again at 14,000×*g* for 20 min. Then 100 μL of 0.05 M iodoacetamide in UA buffer was added, and the samples were incubated at room temperature for 20 min in darkness. After 10 min centrifugation at 14,000×*g*, 100 μL UA buffer was added and centrifuged at 14,000×*g* for 15 min. This step was repeated twice. Subsequently, 100 μL ABC buffer (0.05 M NH_4_HCO_3_ in water) was added to the filters and the samples were centrifuged for 10 min at 14,000×*g*. This step was also repeated twice as reported previously [32]. Finally, 2 μg of trypsin (Promega, USA) in 40 μL ABC buffer was added to each filter, and incubated overnight at 37°C. The resulting peptides were collected by centrifugation at 14,000×*g* for 10 min and then the filters were rinsed with 40 μL 10× DS buffer (50 mM triethylammonium bicarbonate at pH 8.5) and centrifuged again [32]. The peptide content was estimated by absorbance at 280 nm using an extinction coefficient of 1.1 for a 0.1% (g/100 mL) solution that was calculated based on the frequency of tryptophan and tyrosine in vertebrate proteins [33].

### Isobaric tag for relative and absolute quantitation (iTRAQ) labeling and phosphopeptide enrichment

Equal amounts of the trypsin-digested samples from the four groups were labeled as CK-114, 1 h-115, 3 h-116 and 6 h-117 utilizing the iTRAQ Multiplex (4-plex) kit (Applied Biosystems, USA); three biological replicates were performed. For the process of enriching phosphopeptides, each labeled peptide mixture was concentrated in a vacuum concentrator and resuspended in 500 μL buffer containing 2% glutamic acid, 65% acetonitrile, and 2% trifluoroacetic acid. TiO_2_ beads (500 μg, GL Sciences, Japan) were added and the mixture was agitated for 40 min then centrifuged for 1 min at 5000×*g* [34]. The supernatant from the first centrifugation was mixed with another 500 μg of TiO_2_ beads and agitated and centrifuged as before. The two bead collections were combined and washed three times with 50 μL of 30% acetonitrile, 3% TFA and then three times with 50 μL of 80% acetonitrile, 0.3% trifluoroacetic acid to remove the remaining non-adsorbed material. Finally, the phosphopeptides were eluted with 50 μL of elution buffer (40% acetonitrile, 15% NH_4_OH) followed by lyophilization and mass spectrometry (MS) [35].

### Nano-liquid chromatography

Each iTRAQ sample (5 μL) was mixed with 15 μL 0.1% trifluoroacetic acid (v/v), and subsequently a 5 μL of the mixture was loaded onto a C18-reversed phase column (15 cm long, 75 μm inner diameter, RP-C18 3 μm, packed in house) in buffer A (0.1% formic acid) and separated with a linear gradient of buffer B (80% acetonitrile and 0.1% formic acid) at a flow rate of 250 nL/min (controlled by IntelliFlow technology; Applied Flow Technology, USA) over 240 min on a Q Exactive MS (Thermo Finnigan,USA) equipped with Easy nLC (Thermo Fisher Scientific, USA). The peptides were eluted with a gradient of 0–55% buffer B from 0 to 220 min, 55–100% buffer B from 220 to 228 min, and 100% buffer B from 228 to 240 min.

### Tandem MS analysis

We analyzed the peptides in positive ion mode and acquired the MS spectra using a data-dependent, top10 method, dynamically choosing the most abundant precursor ions from the survey scan (300–1800 m/z) for subsequent high-energy collisional dissociation (HCD) fragmentation [36]. Determination of the target value was based on predictive automatic gain control(pAGC). The dynamic exclusion duration was 25 s. Survey scans were acquired at a resolution of 70,000 at m/z 200 and resolution for HCD spectra was set to 17,500 at m/z 200. Normalized collision energy was 29 eV and the under fill ratio, which specifies the minimum percentage of the target value likely to be reached at maximum fill time, was defined as 0.1%. The instrument was operated with the peptide recognition mode enabled. Each iTRAQ experiment was analyzed at least three times.

### Data analysis

Mascot 2.2 (Matrix Science) and Proteome Discoverer 1.3 software (Thermo Scientific, USA) were used for identification and quantitative analysis [37]. The reference sequences in the Cotton Genome Project (CGP) database derived from the *Gossypium raimondii* genome include 40,976 non-redundant protein-coding genes sequences and 39316 non-redundant peptide sequences (ftp://public.genomics.org.cn/BGI/cotton/Annotation/G.raimondii.pep.fasta.gz, updated to 07-23-2012, downloaded on 12-05-2012) [38]. A q-value is the minimal false discovery rate at which the identification is considered correct. The dataset was filtered to give a <1% false discovery rate (q-Value ≤ 0.009, Table S1) at peptide level using the target decoy method [39]. The phosphorylated sites on the identified peptides were assigned again using the PhosphoRS algorithm, which calculated the possibility of the phosphorylated site from the spectra matched to the identified peptides. According to the instruction of the software, for each phosphorylation site(s) on all phosphopeptides, PhosphoRS probabilities were set above 75%, indicating that the site is truly phosphorylated, and PhosphoRS scores were set above 50, indicating a good peptide spectral match [40], [41]. Proteome Discoverer 1.3 software was used to extract the peak intensity within 20 ppm of each expected iTRAQ reporter ion from each analyzed fragmentation spectrum. Only spectra in which all the expected iTRAQ reporter ions (four for HCD in this work) were detected were used for quantification.

The intensity of the reporter ions was used for phosphopeptide quantification. We normalized the phosphopeptide ratios by dividing by the median ratio of all peptides identified. As for the quantitative analysis, the log_2_ fold-change values (Treatment/Control) for each time point were calculated for each phosphopeptide. Only phosphopeptides detected in at least two out of the three biological replicates were used for assessment of significant change. In some cases, the quantitative values of certain time points were not available due to missing phosphopeptide identification in one particular sample or the intensity values failed to pass the cutoff. The t-test was employed to identify significant changes between the control and treatment sample among the three biological replicates. The phosphopeptides that passed t-test with p-value<0.05 were considered to be significantly regulated. We also included the cutoff for the log_2_ fold change values, in which the phosphorylation changes were considered highly significant if the log_2_ value ≥ 0.58 or ≤−0.58 (increasing or decreasing 1.5 fold in phosphorylation activity).

### Protein annotation and classification

To identify the corresponding protein for each phosphopeptide, each phosphopeptide was searched against the CGP peptide database [38]. To overcome the challenge that multiple proteins may share the same peptide, the annotations were confirmed by comparison to the annotation of the top score protein hits from an in-house BLAST search against the CGP peptide database.

Identified phosphoproteins were classified based on: (a) the biological processes of each gene product according to annotations in the CGP database and (b) the gene product subcellular localization predicted using the SherLoc2 web server applying the defaulting settings (http://abi.inf.uni-tuebingen.de/Services/SherLoc2) [21].

## Results and Discussion

### Phosphopeptide identification

We profiled the phosphoproteome of the cotton leaf after exposure to SNP in order to gain insight into plant cellular responses to NO. Approximately 2291 phosphopeptides were identified across the cotton database. Subsequently, we removed redundant and invalid peptides and additionally set the PhosphoRS probabilities above 75% and PhosphoRS scores above 50, according to standard practices, to further select for unique phosphopeptides. Following this selection process, we identified 1315 unique phosphopeptides, collectively containing 1528 non-redundant phosphorylation sites (Table S1). Of these non-redundant phosphorylation sites, 1371 (89.7%) were found to be phosphorylated at serine residues, 145 (9.5%) at threonine, and 12 (0.8%) at tyrosine (Figure S2A). Of the 1315 unique phosphopeptides, 1110 were singly phosphorylated, 198 were doubly phosphorylated, 6 were phosphorylated at three sites, and in only one case the phosphopeptide was phosphorylated at four sites (Figure S2B). The distribution of phosphor-Ser (pS) was found to be consistent with that of rice (89.5%) [22] and Glycine max (89.3%) [28]. Moreover, the distribution of phosphor-Thr (pT) was similar to Arabidopsis (9.9%) [22]. However, the abundance of tyrosine phosphorylation in cotton (0.8%, this study) was slightly lower than in Arabidopsis (2.4%) [22], Medicago (1.3%) [42] and rice (1.6%) [22]. On the other hand, Laurent et al. reported a proportion of pY in soybean of only 0.48% [28]. Additionally, within the same species (e.g. Arabidopsis), the distribution of the phosphor-amino acids was not always consistent [22], [43]. These differences may be attributed to differences in methodology (e.g. phosphopeptide enrichment and/or LC-MS) or in the biological system, where each cell type, tissue and organism has a unique phosphoproteome profile.

### Phosphorylation site motifs

To determine the potential consensus sequences for cotton, the motifs of the 1528 non-redundant cotton phosphorylation sites were extracted using the Motif-X program (version v1.2 10.05.06; http://motif-x.med.harvard.edu/motif-x.html) with default parameters [44], [45]. Because *Gossypium raimondii* Protein Fasta data has a size surpassing the upload restrictions to 10 MB, the database included in 20,000 sequences selected randomly from *Gossypium raimondii* Protein Fasta data was used as the background in Motif-X. Only confidently identified phosphorylation sites with Ascore >19 were used in analysis. All 25 of the phosphorylation site motifs identified in cotton are presented in Table 1. Serine was phosphorylated in 20 of the motifs, threonine in 4, and tyrosine in 1. Table 1 also lists the phosphorylation site motifs that contained at least one fixed position aside from the central phosphorylated residue.

**Table 1:** Cotton phosphorylation site motifs detected in our study compared with motifs identified in Arabidopsis and PhosphoMotif Finder.

No.	Cotton*	Arabidopsis	PhosphoMotif Finder Database
1	……**S**PR….		
2	…..R**S**.S….		
3	..SP..**S**……		
4	……**S**…SP.		
5	……**S**P.R…		
6	…RS.**S**……		
7	……**S**PK….		
8	……**S**.SP…		
9	R..S..**S**……		
10	..S…**S**P…..		
11	…..G**S**P…..		
12	…RQ.**S**……	N	N
13	……**S**DDE…		
14	.L.R..**S**……		
15	….SP**S**……		
16	……**S**D.E…		
17	…SP.**S**……		
18	……**S**R..S..		
19	……**S**D.D…		
20	…..D**S**.E….	N	N
21	…..R**T**.S….		
22	……**T**….SP	N	N
23	….SP**T**……		
24	…SP.**T**……	N	N
25	….P.**Y**……		

*The phosphorylated residues are indicated in bold and underlined, and (.) indicates any amino acid. N indicates the motif was not found.

With the purpose of investigating the specificity of the identified motifs to cotton, these motifs were compared with literatures [46], [47] as well as in the PhosPhAt 4.0 database of Arabidopsis phosphorylation site motifs (http://phosphat.mpimp-golm.mpg.de/phosphat.html) [48] and Human protein reference database (HPRD) [49]. All the phosphorylation motifs from Arabidopsis and cotton are shown in Table S2A and the information of cotton peptide sequence can be found in Table S2B. Among the 25 motifs identified in cotton, 4 of the motifs (RQxS, DSxE, TxxxxSP and SPxT) were neither present in the Arabidopsis database nor present in the Human protein reference database (HPRD) (Table 1).

### Phosphoprotein identification

To identify the corresponding proteins for each phosphopeptide, the phosphopeptides in our dataset were individually searched against the CGP peptide database. As expected, the longer peptides tended to identify single proteins, but the shorter peptides tended to be shared by multiple proteins. To overcome this potential biasing aspect, the annotations of all of phosphopeptides were confirmed by comparing them to the annotation of the top score protein hits in CGP peptide database. From 1528 unique phosphopeptides, 1020 unique phosphoproteins were identified and all of sequence information could be found in Table S3. Passing through several steps of protein digestion, phosphopeptide enrichment, fractionation and mass spectrometry analysis, it is possible that for a single protein, two or more phosphopeptide is retained and can be detected in the LC/MS analysis. As much, our results indicated that, among the 1020 phosphoproteins, 81% were represented by a single phosphopeptide, 13.2% by two phosphopeptides, 3.7% by three phosphopeptides, and 2.1% by more than three phosphopeptides (Figure S3).

**Figure 1:**
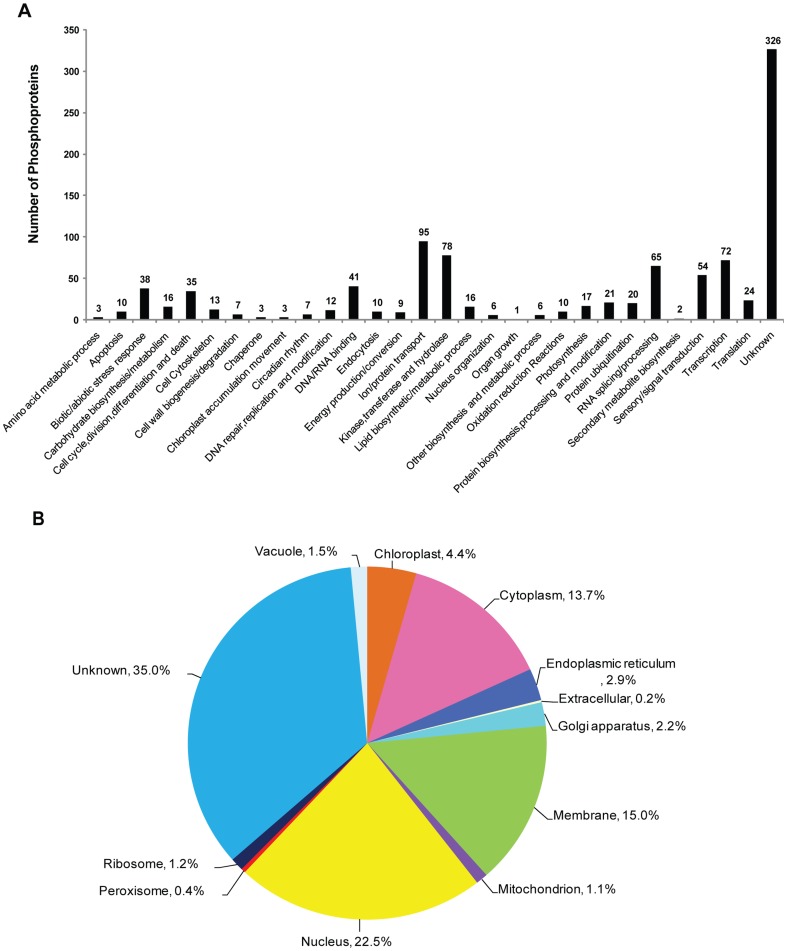
Functional classifications (A) and protein subcellular locations (B) of all 1020 phosphoproteins.

To obtain a general overview of the roles of phosphorylation in cotton, we next analyzed the biological functions/pathways and cellular localization of the 1020 phosphoproteins. The proteins were classified into 30 different categories based on their predicted functions, covering a wide range of pathways. The largest functional groups were ion/protein transport–related proteins, various enzymes (including kinases, transferases, and hydrolases), RNA splicing/processing, transcription, and sensory and signal transduction, accounting for 9.3, 7.7, 7.1, 6.4 and 5.3% of the 1020 proteins, respectively (Figure 1A). No function/pathway could be assigned to 32.0% of the phosphoproteins identified. For the subcellular localization analysis, these 1020 phosphoproteins were located in 12 different cellular compartments, including primarily the nucleus (22.5%), membranes (15.0%), cytoplasm (13.7%) and chloroplasts (4.4%). Exact subcellular localization information was not available for 35.0% of the proteins (Figure 1B).

### Quantitative analysis of protein phosphorylation changes in response to NO

**Figure 2:**
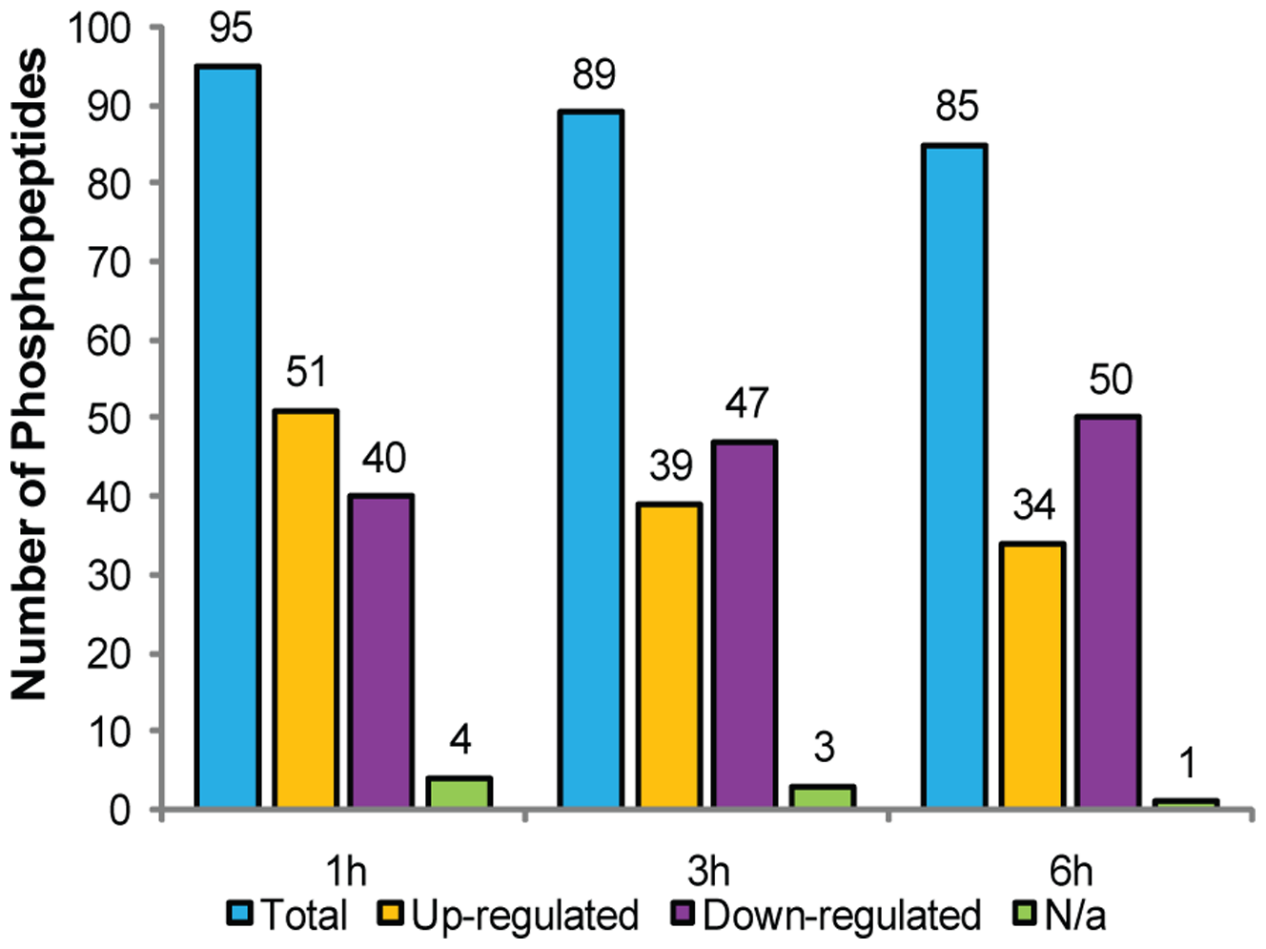
Analysis of the 183 phosphopeptides whose levels changed after exposure to SNP. N/a, not applicable, indicates that the phosphopeptides were not detected in this group and were only expressed in other treatment groups.

The fold change (treatment/control) for every phosphopeptide at each time point was calculated, and the amounts of 183 unique phosphopeptides derived from 167 phosphoproteins were found to change significantly (>1.5 fold change and P-value ≤ 0.05) after SNP exposure (Table S4). The total number of differentially expressed phosphopeptides was 95, 89, and 85 at 1, 3, and 6 h, respectively (Figure 2). A decreasing trend was observed for the number of upregulated phosphopeptides, which decreased from 53.7% in the 1 h group to 40.0% in the 6 h group. Conversely, the number of downregulated phosphopeptides increased gradually from 42.1% in the 1 h group to 52.8% in the 3 h group and 58.8% in the 6 h group. Quantitative analysis revealed that most of the phosphopeptide changes occurred rapidly, i.e., within an hour of first exposure to SNP, which is consistent with the initiation of signal transduction and is similar to other phosphoproteomics studies [28]. As such, we hypothesized that, for phosphoproteins regulated by NO, we should see a shift from the phosphorylated to the dephosphorylated forms as the treatment continued.

**Figure 3:**
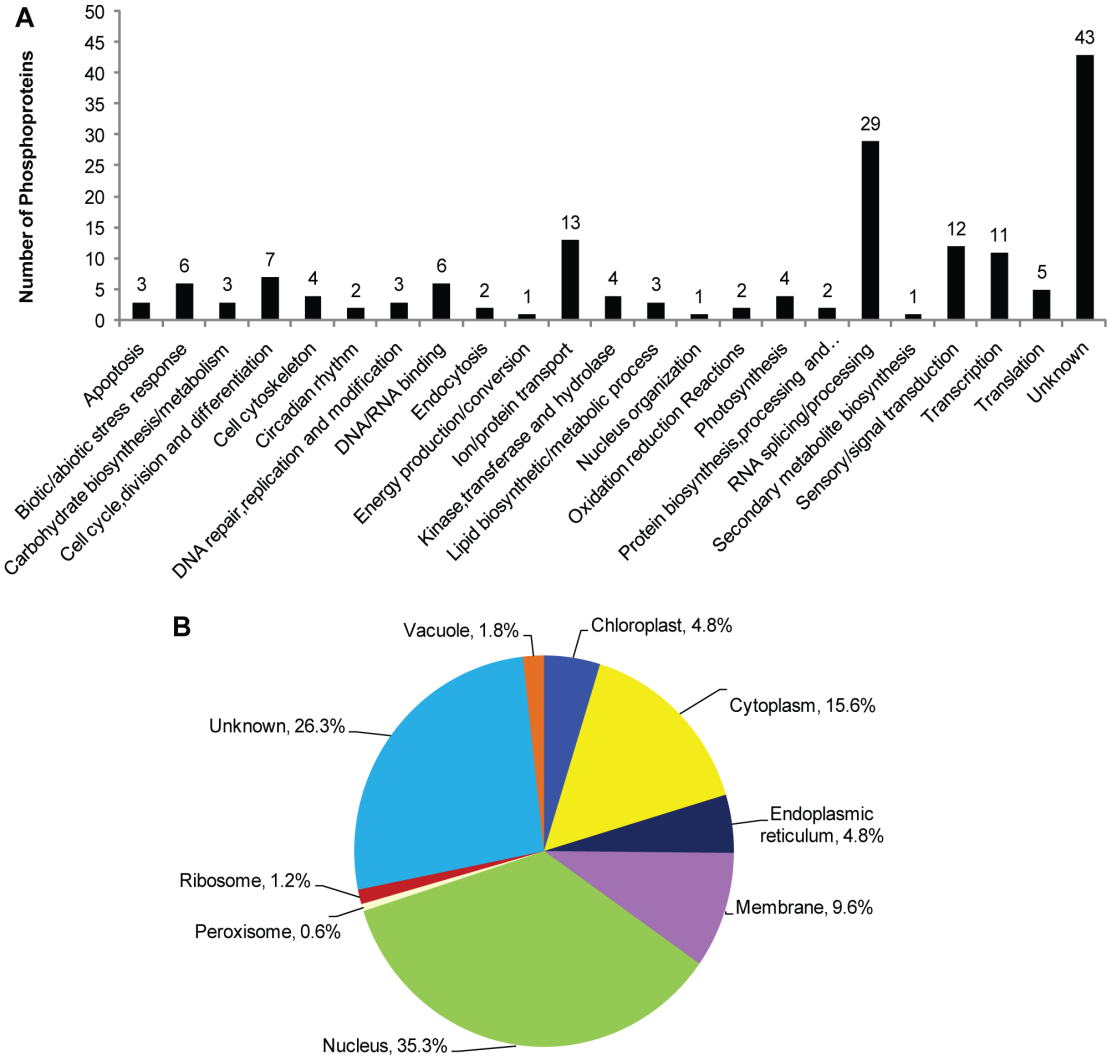
Functional classifications (A) and protein subcellular locations (B) of the 167 phosphoproteins.

The 167 phosphoproteins whose phosphopeptides levels changed significantly in response to NO were classified into 23 functional categories based on their predicted biological function (Figure 3A). Except for the unknown proteins (25.7%), the largest functional groups were RNA splicing or processing (17.4%), ion or protein transport (7.8%), sensory or signal transduction (7.2%), and transcription (6.6%). Compared with the total dataset of all the non-redundant phosphoproteins identified, our results suggest that these functional categories are the most important in terms of response to NO. Correspondingly, the largest subcellular location groups were the nucleus (35.3%), unknown (26.3%), the cytoplasm (15.6%), and membranes (9.6%) (Figure 3B).

### Ion/Protein transport proteins

Many (95) of the cotton phosphoproteins that we identified are involved in ion/protein trafficking processes, such as potassium, ammonium, sucrose, and peptide transport, or are transporter proteins themselves (i.e., aquaporin and ABC transporter). ATPases are post-translationally regulated by complex mechanisms, including phosphorylation [50], [51]. In our present study, the abundance of phosphopeptides corresponding to 16 ATPases was found to be altered, including 2 V-type and 9 plasma membrane-type phosphopeptides. These results suggest that NO regulation of transporters by phosphorylation may play a role in quick and reversible activating/inactivating transport channels, leading to rapid changes in water potential and turgor pressure that are necessary for plant growth and stimulus response.

### Miscellaneous enzymes

As shown in Figure 1A, a considerable number (78) of the cotton phosphoproteins identified were classified as “miscellaneous enzymes”, including kinases, transferases, and hydrolases. However, only 4 of these proteins, all kinases, appeared in our quantitative phosphoproteomic data (Figure 3A). We hypothesize that changes in the abundance of these proteins might reflect a coordinated activity of one or more kinase cascades involved in the NO-response process. How these proteins fit into the context of NO signaling and their relationships with other well-known receptor and protein kinases need to be further explored in future studies.

### Proteins involved in DNA/RNA binding and transcription

Among the 72 transcription-related phosphoproteins identified, the content levels of only 11 significantly changed in response to SNP (Figure 3A), including transcription initiation/elongation factors, transcriptional co-repressors, and some sequence-specific transcription factors. DNA/RNA-binding proteins have been identified in several phosphoproteomic studies [28], [42]. Similarly, we found 41 phosphoproteins in cotton that appear to be involved in a variety of DNA/RNA processes (Figure 1A). In addition, we found 7 differentially expressed phosphoproteins involved in DNA/RNA binding, including RNA-binding motif protein (Cotton_D_gene_10033131) expressed predominantly in spematocytes [52], HMG1/2-like protein (Cotton_D_gene_10035211) containing 1HMG box DNA-binding domain that belongs to the HMGB family which was regulated by darkness and circadian rhythm [53], uncharacterized RNA-binding protein (Cotton_D_gene_10011862) and a number of predicted proteins (Table S1). We speculate that the phosphorylation and the presence of differential phosphorylation of these proteins may be essential for the rapid responses to NO treatment.

### Phosphorylation changes of protein in response to plant hormones

The 187 phosphopeptides, corresponding to the 167 identified NO-responsive phosphoproteins, were placed into appropriate signaling pathways to better understand which physiological processes in cotton are regulated by NO. The signaling pathways analysis was carried out using KEGG Automatic Annotation Server (http://www.genome.jp/tools/kaas/) with the default settings.

Ethylene-insensitive protein 2 (EIN2) acts downstream of ethylene receptors and upstream of ethylene-regulated transcription factors and is involved in regulating early leaf senescence caused by NO deficiency [54], [55]. We found that the levels of phosphorylated EIN2 (Cotton_D_gene_10027821) increased gradually after SNP exposure and were significantly higher at 6 h. Levels of another negative regulator in the ethylene response pathway, the serine/threonine-protein kinase CTR1 (Cotton_D_gene_10011591), were also increased in response to NO as early as 1 h after treatment began. In our previous study, we found that NO is involved in regulating ACS [21], a synthase catalyzing the synthesis of a precursor (1-Aminocyclopropane-1-carboxylic acid) in the ethylene biosynthesis pathway. Here, we found that NO appears to regulate the phosphorylation of EIN2 and CTR1, central factors in the signaling pathways regulated by ethylene. Thus, we postulate that NO regulates not only the biosynthesis of ethylene but also the ethylene-signaling pathway by modulating phosphorylation of EIN2 and CTR1.

In our study, NO also appeared to affect the signal transduction pathways of other plant hormones, such as cytokinine and ABA (Figure S4). Inhibitors of NO-synthase have been shown to inhibit expression from the cytokinine-responsive *ARR5* promoter [56]. The two-component response regulator ARR2 is also a transcriptional activator involved in cytokinine signaling and is phosphorylated in response to cytokinine [57], [58]. In the current paper, we found that levels of phospho-ARR2 (Cotton_D_gene_10029097) were markedly reduced in the 1 and 3 h treatment groups. This decrease is likely related to the NO-regulated cytokinine signaling pathway. Protein phosphatase 2C (PP2C) 37 is a major negative regulator of ABA responses during seed germination and cold acclimation [59], [60]. We also found that the levels of phosphorylated PP2C 37 (Cotton_D_gene_10021530) were increased at both the 1 and 3 h time points. Moreover, NO mediates the induction of ABA biosynthesis involved in oxidative stress tolerance in maize (*Zea mays*) [61], [62]. Additionally, PP2C inhibits SnRK2, a vital kinase in the regulation of ABA-induced gene expression, thus inhibiting activation of the ABA pathway [25]. Given this extensive cross-talk between NO and ABA, it is plausible that the apparent dephosphorylation of PP2C 37 that we observed may play a role in ABA signal transduction regulated by NO.

Many components of the auxin efflux (but not influx) system are activated by phosphorylation [28], [63]. In this study, the phosphopeptide levels of one auxin-related protein were significantly altered in response to NO. Furthermore, we found that a phosphoprotein required for auxin influx-facilitator (AUX1) polar trafficking (Cotton_D_gene_10018241) was significantly increased 1 h after SNP treatment. Thus, it appears that increases in phosphorylation of both efflux and influx carriers facilitate auxin transport in cotton.

### Phosphoproteins involved in RNA metabolism

Our study also identified numerous phosphoproteins involved in RNA metabolism, including components of the spliceosome, mRNA surveillance, and RNA transport proteins. Specifically, 21 phosphoproteins, corresponding to 13 KEGG orthology entries involved in spliceosome activity were detected (Figure S5), 8 of which were SR proteins (serine/arginine-rich splicing factors; Cotton_D_gene_10019600, Cotton_D_gene_10022365, Cotton_D_gene_10039654, Cotton_D_gene_10032493, Cotton_D_gene_10026606, Cotton_D_gene_10024595, Cotton_D_gene_10024595, and Cotton_D_gene_10030715). SR proteins belong to a highly conserved family of splicing regulators involved in constitutive and alternative splicing in response to stress and hormones [64] and have been shown to be phosphorylated in both animals and plants [65]–[67]. Moreover, kinase-protein interactions related to pre-RNA splicing are modulated by the phosphorylation of SR proteins [68]. The 8 SR proteins we detected were extensively phosphorylated at serine residues in their serine/arginine-rich domains (Table S1), similar to the phosphorylation of SR proteins in Arabidopsis [69].

**Figure 4:**
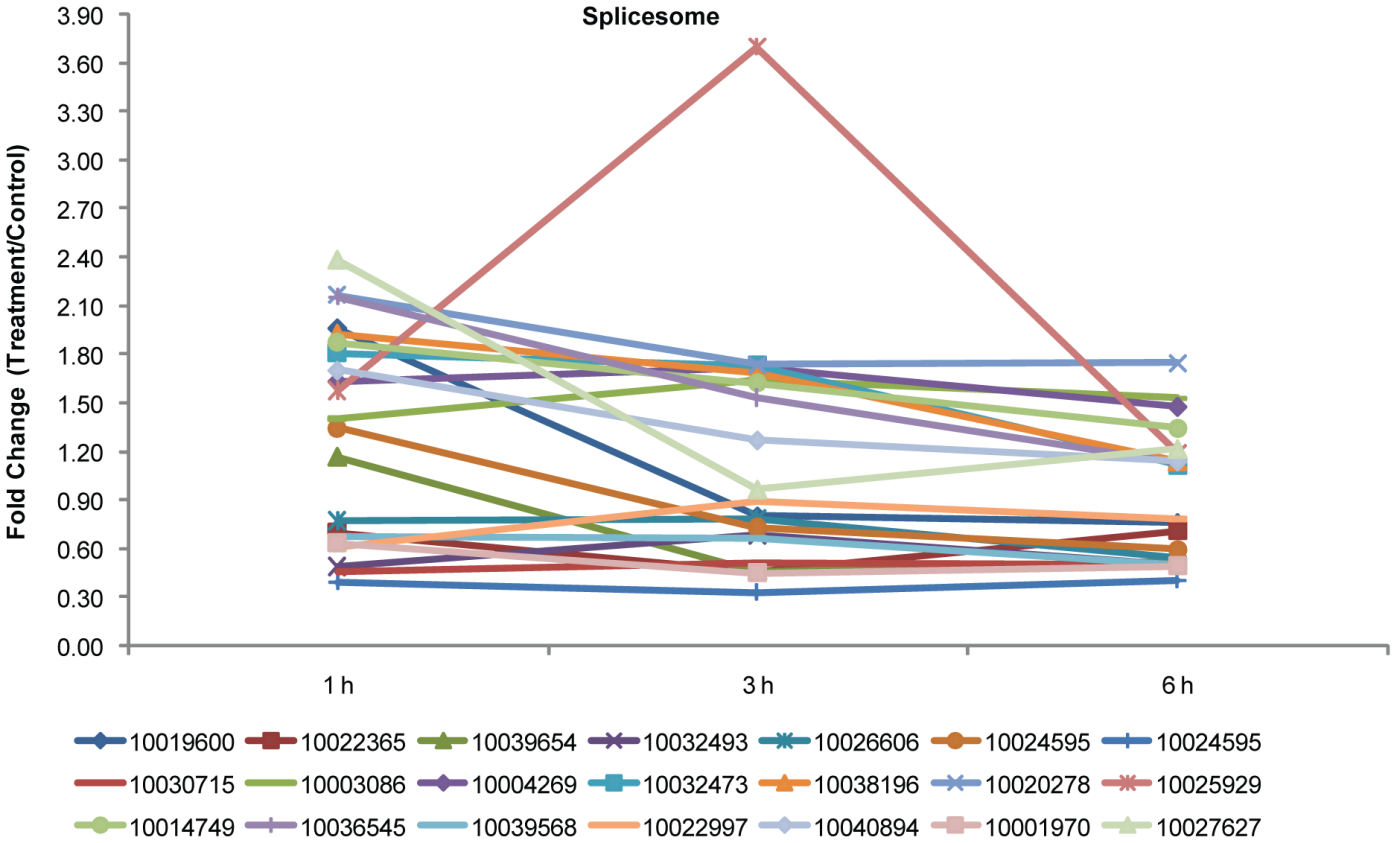
Fold changes of the phosphopeptides involved in spliceosome significantly altered following SNP. Protein identification numbers refer to the CGP database. To make the figure easier to read, the protein ID is represented only by the numbers in the key (i.e., remove “Cotton_D_gene_”).

The phosphorylation of some helicases in humans and Arabidopsis has been shown to regulate pre-mRNA splicing [69]–[71]. Our analysis identified two ATP-dependent RNA helicases (Cotton_D_gene_10003086, Prp22; Cotton_D_gene_10004269, Prp2), suggesting that phosphorylation of the cotton homologs may also function in splicing. Small nuclear RNA conformation and the phosphorylation of related proteins strongly affect 3′-splice site recognition and catalytic activation of the spliceosome [72], [73]. We found that the levels of phosphorylated forms of two small nuclear ribonucleoproteins (Cotton_D_gene_10032473, Prp3; and Cotton_D_gene_10038196, Prp4) and one small nuclear ribonucleoprotein helicase (Cotton_D_gene_10020278, Bn2) increased in response to NO within 3 h of exposure to SNP. In addition, the phosphorylated form of a pre-mRNA processing factor (Cotton_D_gene_10025929, FBP11), the nuclear cap-binding protein (Cotton_D_gene_10014749, CBP80/20), and a nuclear apoptotic chromatin-condensation inducer (Cotton_D_gene_10036545, ACINUS) also increased 1 h and 3 h after SNP treatment began. The levels of phosphorylated U2-associated protein (Cotton_D_gene_10039568, SR140) and RNA-binding protein (Cotton_D_gene_10022997, HnRNPs) also decreased to varying degrees. We also found three splicing factors in this pathway (Cotton_D_gene_10040894, SF36; Cotton_D_gene_10001970, U2AF; Cotton_D_gene_10027627, U2AF) that are involved in phosphorylation in response to NO, indicating a direct link between NO signaling and spliceosome regulation through phosphorylation and dephosphorylation of related proteins. Except for a special pattern that sharp increase followed by sharp decrease (Cotton_D_gene_10001970), the changes in the other 20 spliceosome-related phosphoproteins could be categorized as two distinct patterns–a gradual decline in phosphorylation followed by a sharp increase or a stable decline from 1 to 6 h (Figure 4).

**Figure 5:**
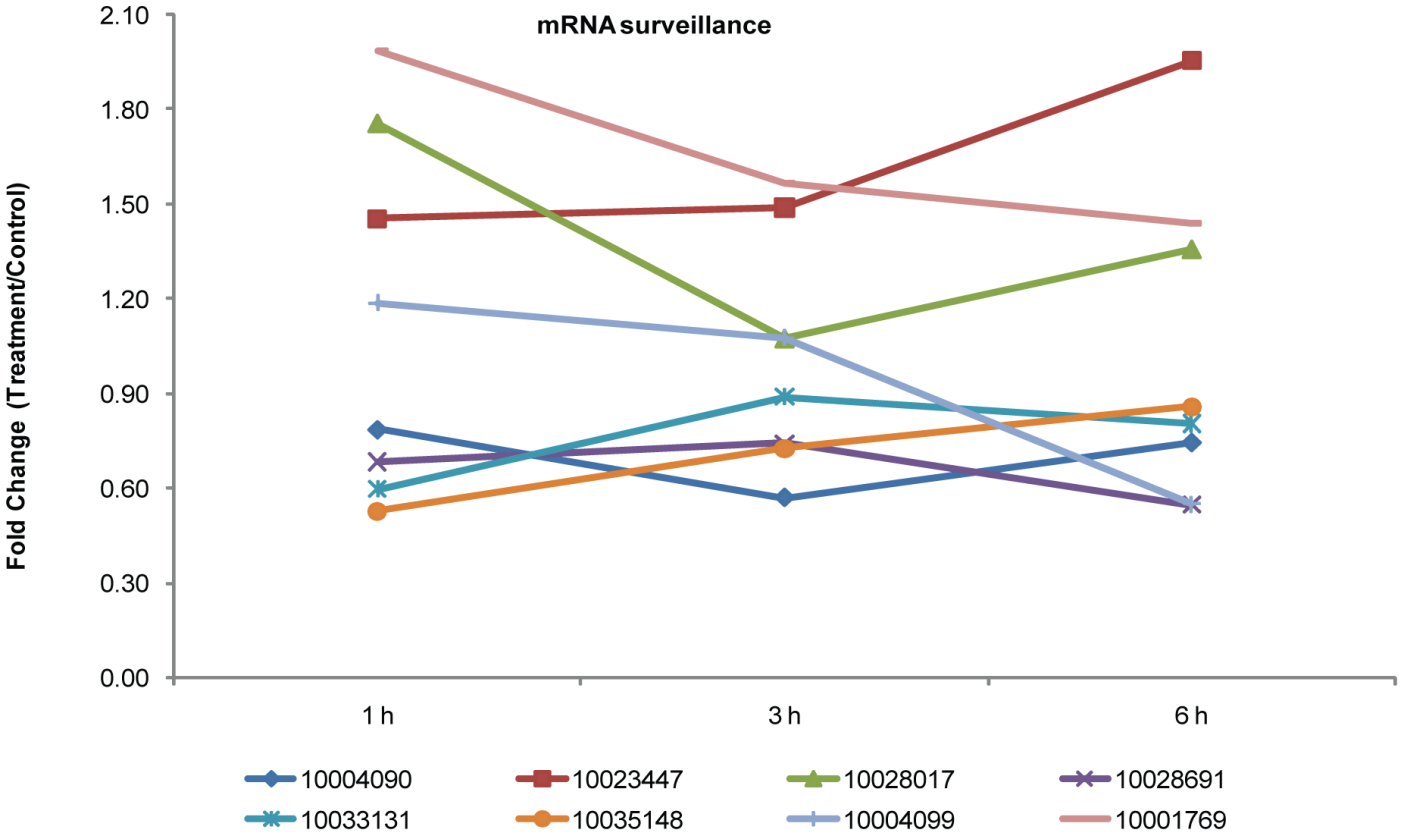
Fold changes of the phosphopeptides involved in mRNA surveillance significantly altered following SNP. Protein identification numbers refer to the CGP database. To make the figure easier to read, the protein ID is represented only by the numbers in the key (i.e., remove “Cotton_D_gene_”).

The mRNA surveillance pathway is a quality-control mechanism that detects and degrades abnormal mRNAs. Upf3, together with Upf1 and Upf2, detects the presence of premature termination codon and instigates decapping and rapid exonucleolytic digestion of the mRNA [74]. The dephosphorylation of hUPF1 by PP2A, contribute to the remodeling of the mRNA surveillance complex [75]. We found that Upf3 (Cotton_D_gene_10004090, Upf3) levels were higher at 3 h in the treated samples than at 1 or 6 h, and the levels of the phosphorylated forms of three PP2A subunits (Cotton_D_gene_10023447, PP2A; Cotton_D_gene_10028017, PP2A; and Cotton_D_gene_10028691, PP2A) also changed over time. In addition, levels of the phosphorylated RNA-binding motif protein (Cotton_D_gene_10033131, CPSF6/7), heterogeneous nuclear ribonucleoprotein (Cotton_D_gene_10035148, Musashi), cleavage and polyadenylation specificity factor (Cotton_D_gene_10004099, CPSF4) and serine/threonine-protein kinase (Cotton_D_gene_10001769, SMG1) also changed significantly in response to NO (Figure 5, Figure S6). The changes in these proteins are consistent with the known critical role of the mRNA surveillance pathway in cellular homeostasis.

**Figure 6:**
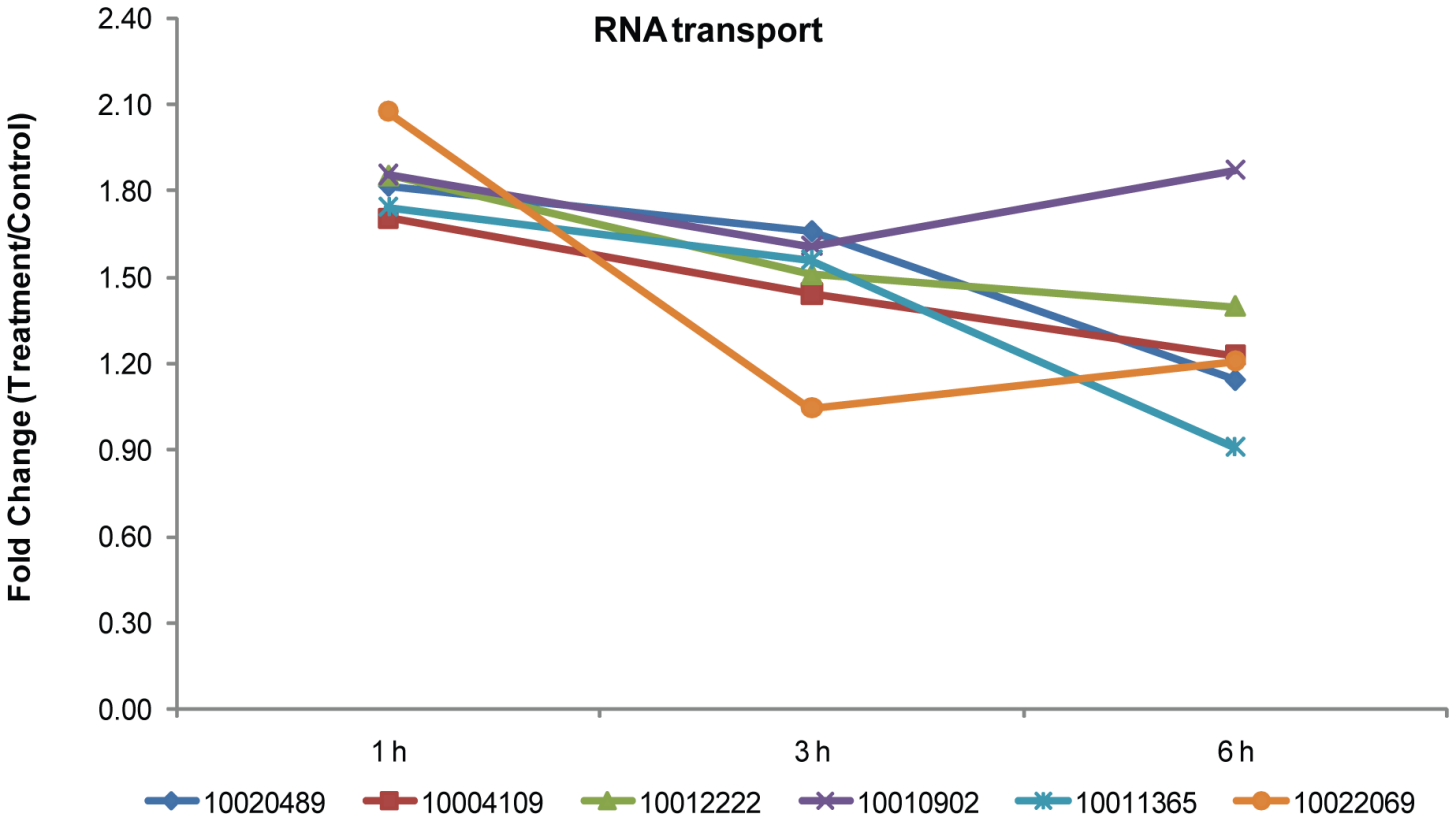
Fold changes of the phosphopeptides involved in RNA transport significantly altered following SNP. Protein identification numbers refer to the CGP database. To make the figure easier to read, the protein ID is represented only by the numbers in the key (i.e., remove “Cotton_D_gene_”).

In addition to the apoptotic chromatin condensation inducer, nuclear cap-binding protein, and Upf3, all mentioned above, we identified another six phosphoproteins involved in RNA transport (Figure S7), including four translation initiation factors (Cotton_D_gene_10020489, eIF3; Cotton_D_gene_10004109, eIF3; Cotton_D_gene_10012222, eIF4G; and Cotton_D_gene_10010902, eIF4A), one ribonuclease (Cotton_D_gene_10011365, RNaseZ), and one unknown protein (Cotton_D_gene_10022069, Nup133). Eukaryotic initiation factor 3 (eIF3) is an essential, highly conserved multiprotein complex that is a key component in the recruitment and assembly of the translation initiation machinery [76]. eIF4F is a protein complex that mediates recruitment of ribosomes to mRNA [77], [78]. The activity of both proteins is regulated by phosphorylation and in turn influences translation, apoptosis, and growth [78], [79]. Analysis of the cotton eIFs revealed that the phosphorylated peptides of all four proteins increased to varying degrees in response to SNP. The pattern for all the RNA transport phosphopeptides showed a rapid increase followed by a steady downward trend (Figure 6).

RNA splicing/processing was the largest group of proteins whose phosphorylation level was significantly affected by NO (Figure 3A). Correspondingly, we found the numerous proteins in the splicesome, mRNA surveillance and RNA transport pathways that were altered noticeably in regards to their phosphorylation status as regulated by NO. These results suggest that the plant mRNA splicing machinery is a major target of phosphorylation or dephosphorylation induced by NO.

### Other phosphorylated proteins

**Figure 7:**
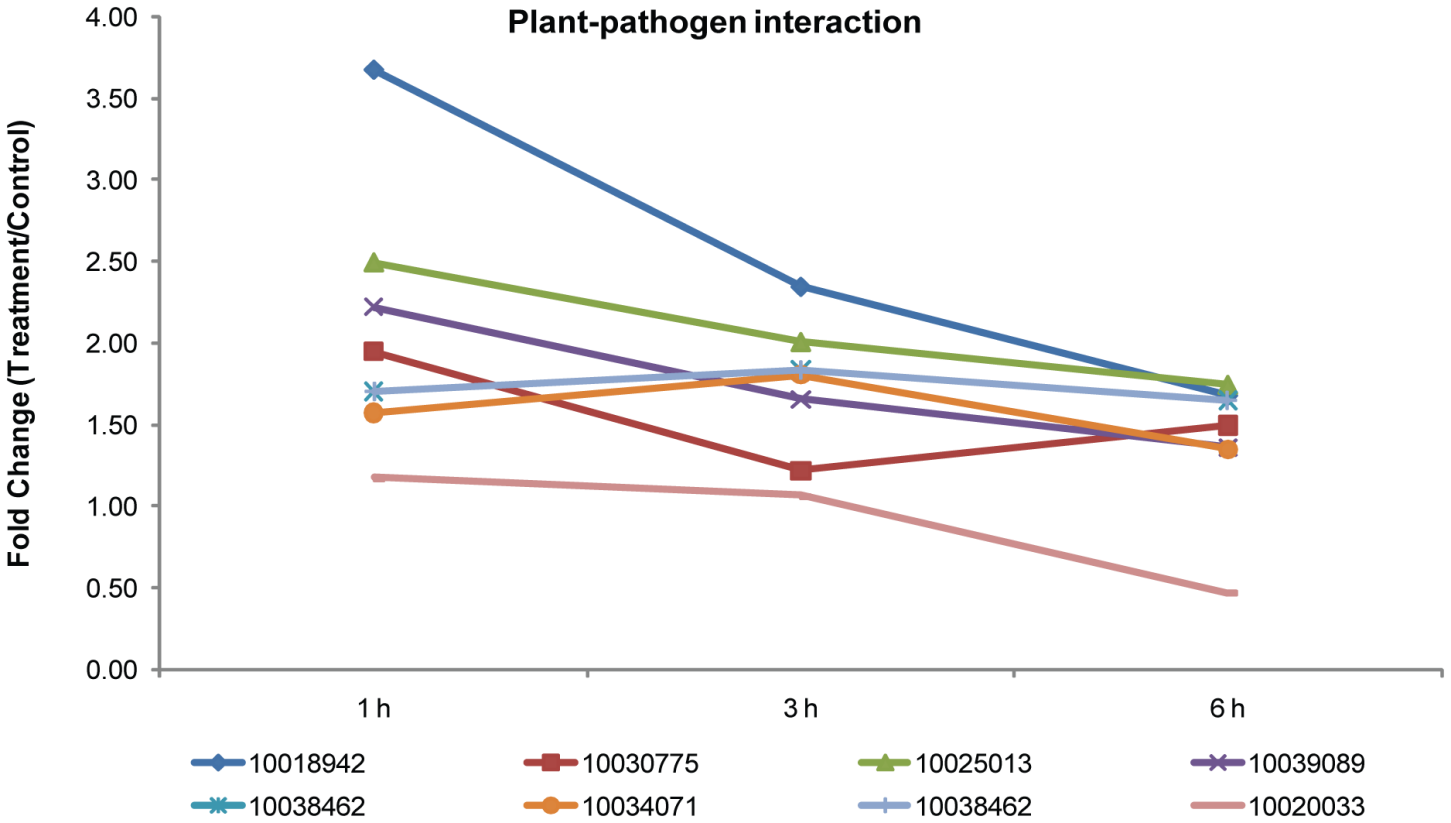
Fold changes of the phosphopeptides involved in plant-pathogen interaction significantly altered following SNP. Protein identification numbers refer to the CGP database. To make the figure easier to read, the protein ID is represented only by the numbers in the key (i.e., remove “Cotton_D_gene_”).

Other phosphoproteins, whose peptide levels changed noticeably in response to NO in the current paper, were found to be involved in protein processing and/or to be related to plant-pathogen interactions [21], [80]. In protein processing, the binding protein, Bip, in the endoplasmic reticulum can recognize improperly folded or assembled and facilitates their refold and reassembly [81], [82]. It has been reported that the transcription of the gene encoding Bip is induced by SNP [21]. Similarly, in the current paper we found that phosphopeptides of Bip (Cotton_D_gene_10017746) were increased significantly after treatment with SNP in the 6 h group. In terms of plant-pathogen interaction proteins, we found that the phosphorylated forms of three heat shock proteins were also dramatically increased by SNP (Cotton_D_gene_10018942, Cotton_D_gene_10030775 and Cotton_D_gene_10025013). A mitogen-activated protein kinase kinase (Cotton_D_gene_10039089, MKK1/2), a calcium-dependent protein kinase (Cotton_D_gene_10038462, CDPK), and a respiratory burst oxidase (Cotton_D_gene_10034071, Rboh) also showed significant upregulation in phosphorylation in varying degrees. On the other hand, an unknown protein (Cotton_D_gene_10020033, CaMCML) showed significant decreases in the phospho-forms (Figure 7, Figure S8). These results indicate that NO regulates plant disease resistance by modulating phosphorylation of the relevant proteins.

It is noteworthy that some key proteins involved in circadian rhythms were also revealed in the phosphoproteome of SNP-treated cotton. The phosphorylated forms of protein LHY (Cotton_D_gene_10035743) and the transcription factor HY5 (Cotton_D_gene_10010118), both important components in clock function, were significantly reduced following treatment with SNP (Figure S9). To our knowledge, this is the first report of a relationship between NO and plant circadian rhythms, especially in terms of phosphorylation regulation. The levels of several other phosphopeptides/phosphoproteins not previously implicated in NO response were significantly also altered by SNP treatment, including translocase of chloroplast 159 (Cotton_D_gene_10024721), stem-specific protein TSJT1 (Cotton_D_gene_10003896), and villin-2 (Cotton_D_gene_10038481). Further exploration of these phosphoprotein targets may provide insight into previously unknown effectors of the NO signaling pathway.

## Conclusions

This study represents one of the first large-scale phosphoproteomic analyses of the NO response in cotton, providing both a global and comparative analysis of protein phosphorylation regulated by NO and further insight into the dynamics of individual phosphorylation sites. Our results reveal phosphorylation site motifs and proteins not previously observed in plants, which may provide insight into NO signaling in cotton as well as plants in general. In addition, the novel plant phosphorylation site motifs could lead to future discovery of novel kinase-substrate interactions. We have submitted the peptide and protein sequences to the plant protein phosphorylation database (P3DB, http://p3db.org/download.php) [31], [83] to help develop future, large-scale cotton proteomic datasets. In this perspective, a better understanding of phosphorylation events may eventually lead to the development of a comprehensive model for the mechanisms of action of NO. Functional analysis of these protein phosphorylation sites by site-directed mutagenesis or reverse genetic approaches will be necessary to further investigate their role in NO signaling.

## Supporting Information

Figure S1 **The effect of NO on cotton growth.** Cotton seeds were treated with 0, 0.05, 0.1, or 0.5 mM SNP for 24 h. Changes in the morphology (A) and chlorophyll content (B) following the treatment of different concentrations of SNP.
(TIF)

Figure S2 **The distribution of phosphorylation sites.** A. Distribution of phosphorylation on serine, threonine, and tyrosine was assessed for all non-redundant localized phosphorylation sites. B. Distribution of single- and multi-phosphorylated peptides showing that the majority of phosphopeptides have only one phosphorylation site.
(TIF)

Figure S3 **The number of phosphopeptides per protein.** The distribution of the number of phosphopeptides per protein is shown for all 1315 unique phosphopeptides.
(TIF)

Figure S4 **The plant hormone signal-transduction pathway derived from KEGG.** Proteins indicated in red were found to be significantly upregulated or downregulated by SNP in our analysis.
(TIF)

Figure S5 **The spliceosome pathway derived from KEGG.** Proteins indicated in red were found to be significantly upregulated or downregulated by SNP in our analysis.
(TIF)

Figure S6 **The mRNA surveillance pathway derived from KEGG.** Proteins indicated red were found to be significantly upregulated or downregulated by SNP in our analysis.
(TIF)

Figure S7 **The RNA transport pathway derived from KEGG.** Proteins indicated red were found to be significantly upregulated or downregulated by SNP in our analysis.
(TIF)

Figure S8 **The plant-pathogen interaction pathway derived from KEGG.** Proteins indicated red were found to be significantly upregulated or downregulated by SNP in our analysis.
(TIF)

Figure S9 **The circadian rhythm pathway derived from KEGG.** Proteins indicated red were found to be significantly upregulated or downregulated by SNP in our analysis.
(TIF)

Table S1 **Phosphopeptide identifications in cotton leaves.**
(XLS)

Table S2 **Phosphorylation motifs identified from cotton (A) and raw peptide data used to extract the given motif of cotton (B).**
(XLS)

Table S3 **Amino acid sequences of 1020 phosphoproteins.**
(XLS)

Table S4 **Quantitative analysis identified significant changes in the content levels of 183 phosphopeptides.**
(XLS)
