# Supplementary figures and images for: Quantitative Phosphoproteomics Analysis of Nitric Oxide–Responsive Phosphoproteins in Cotton Leaf
Source: PLoS One. 2014 Apr 8;9(4):e94261. doi: 10.1371/journal.pone.0094261 (PMC3979775; doi:10.1371/journal.pone.0094261)

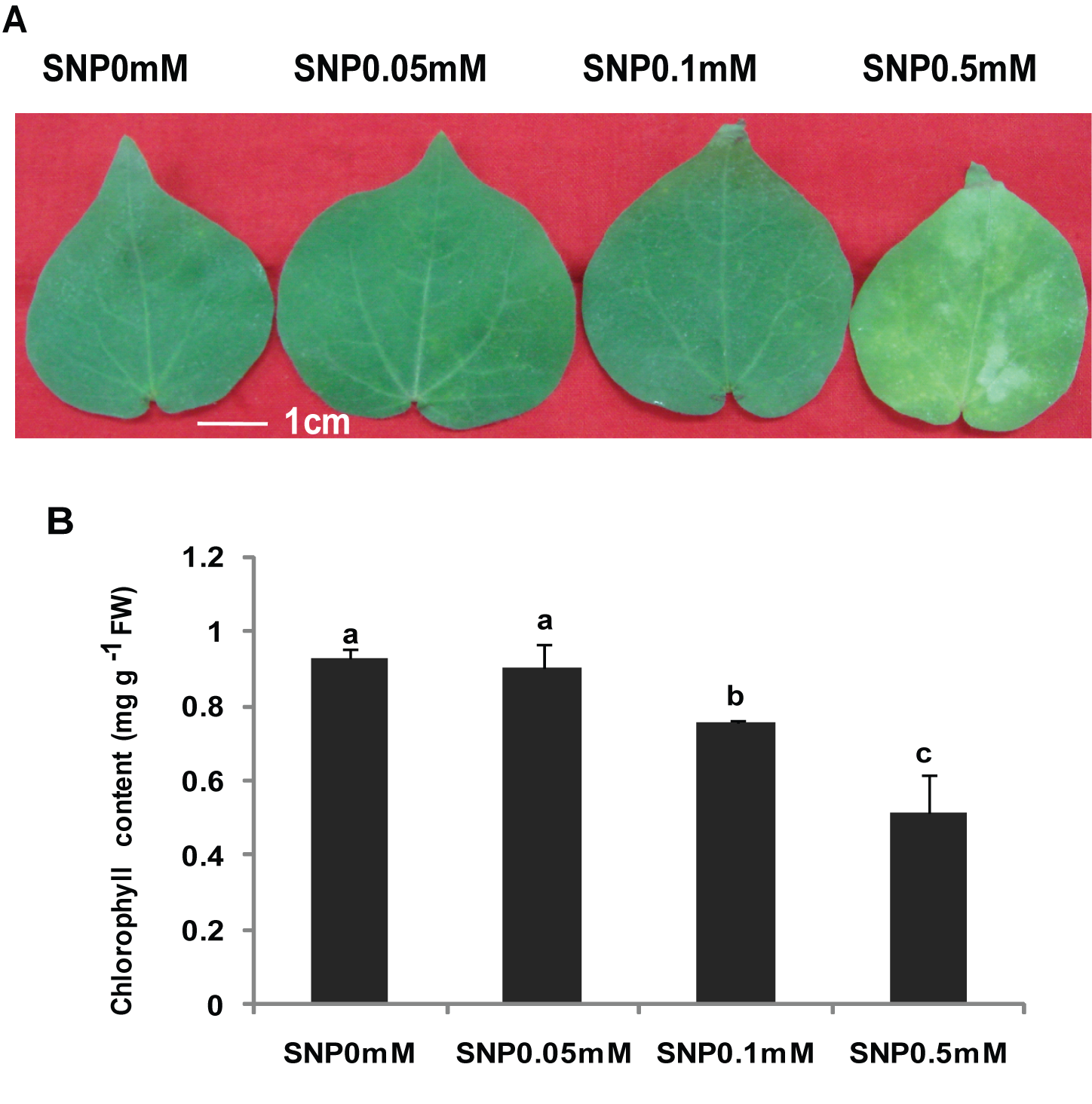

Supplement: Figure S1 — The effect of NO on cotton growth. Cotton seeds were treated with 0, 0.05, 0.1, or 0.5 mM SNP for 24 h. Changes in the morphology (A) and chlorophyll content (B) following the treatment of different concentrations of SNP. (TIF) [file pone.0094261.s001.tif]

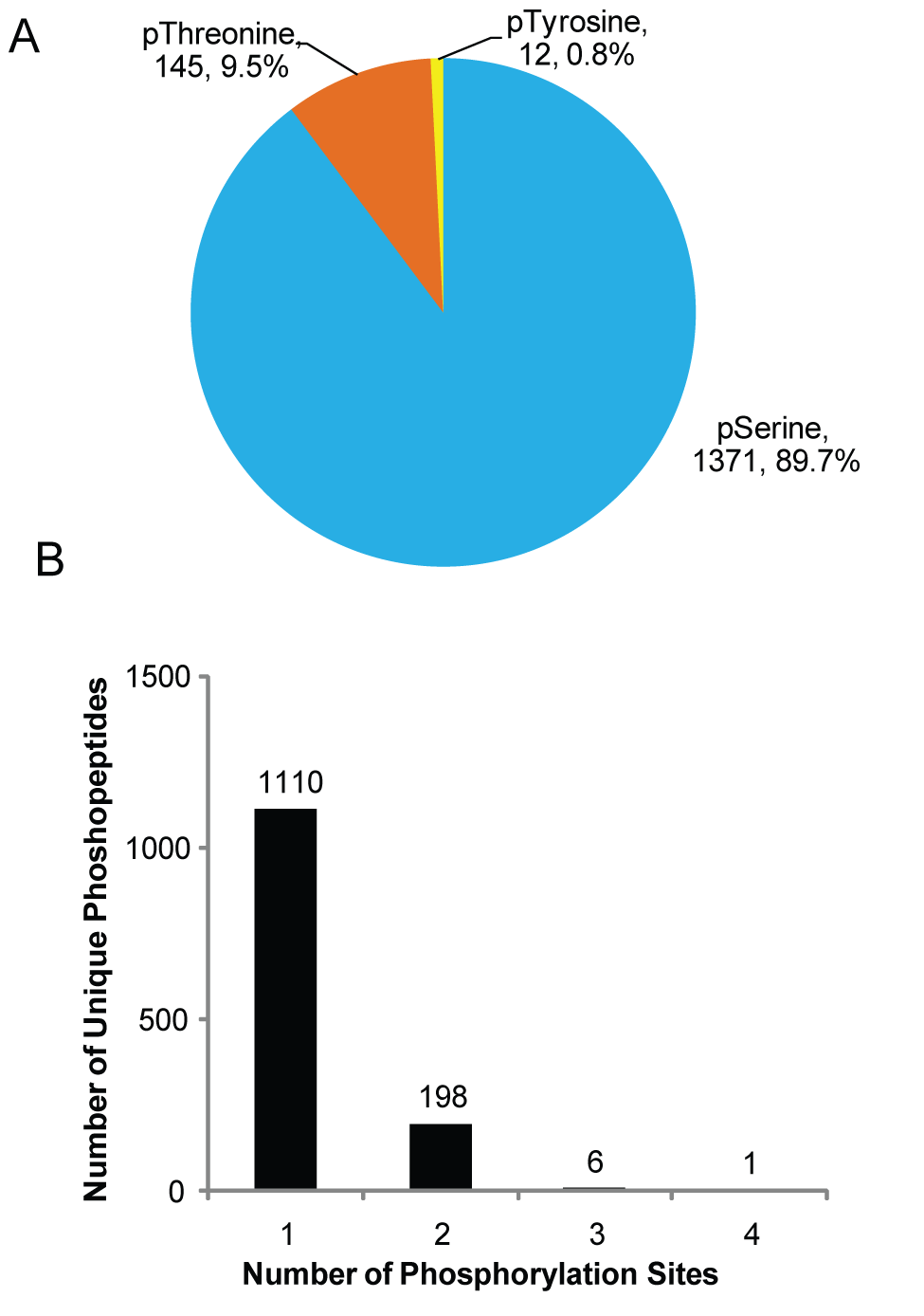

Supplement: Figure S2 — The distribution of phosphorylation sites. A. Distribution of phosphorylation on serine, threonine, and tyrosine was assessed for all non-redundant localized phosphorylation sites. B. Distribution of single- and multi-phosphorylated peptides showing that the majority of phosphopeptides have only one phosphorylation site. (TIF) [file pone.0094261.s002.tif]

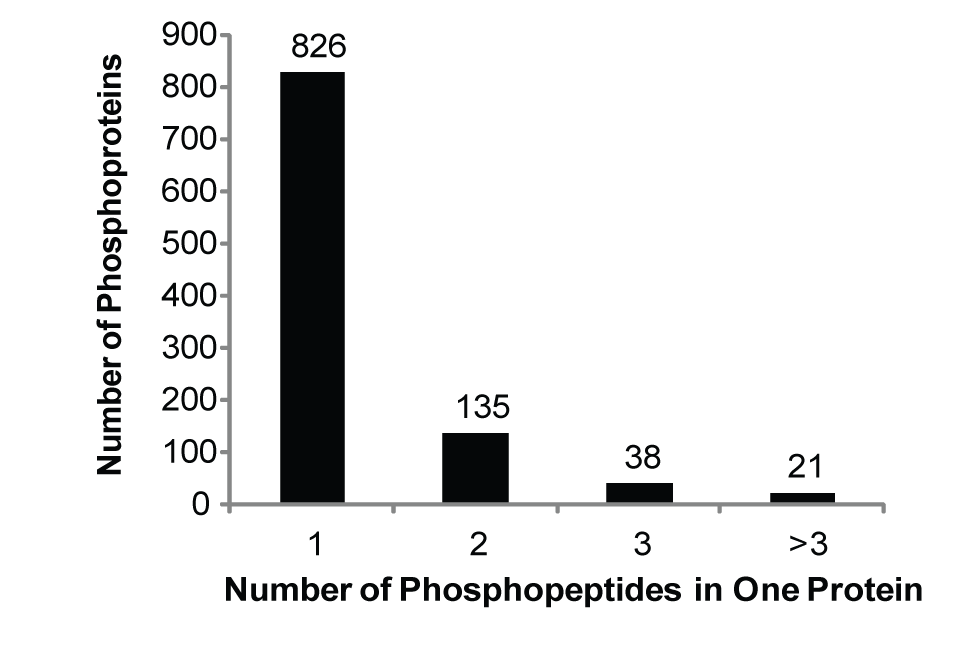

Supplement: Figure S3 — The number of phosphopeptides per protein. The distribution of the number of phosphopeptides per protein is shown for all 1315 unique phosphopeptides. (TIF) [file pone.0094261.s003.tif]

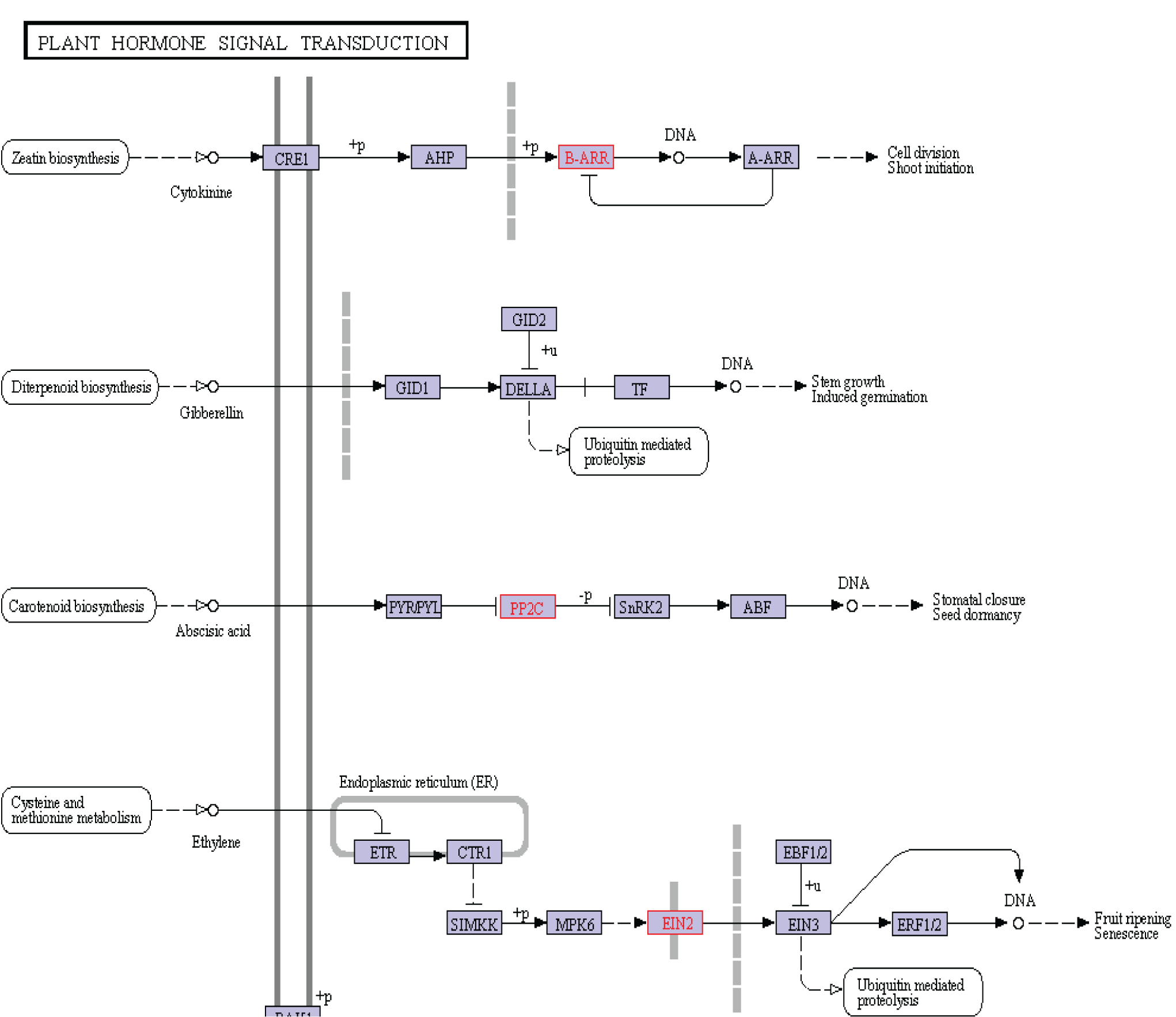

Supplement: Figure S4 — The plant hormone signal-transduction pathway derived from KEGG. Proteins indicated in red were found to be significantly upregulated or downregulated by SNP in our analysis. (TIF) [file pone.0094261.s004.tif]

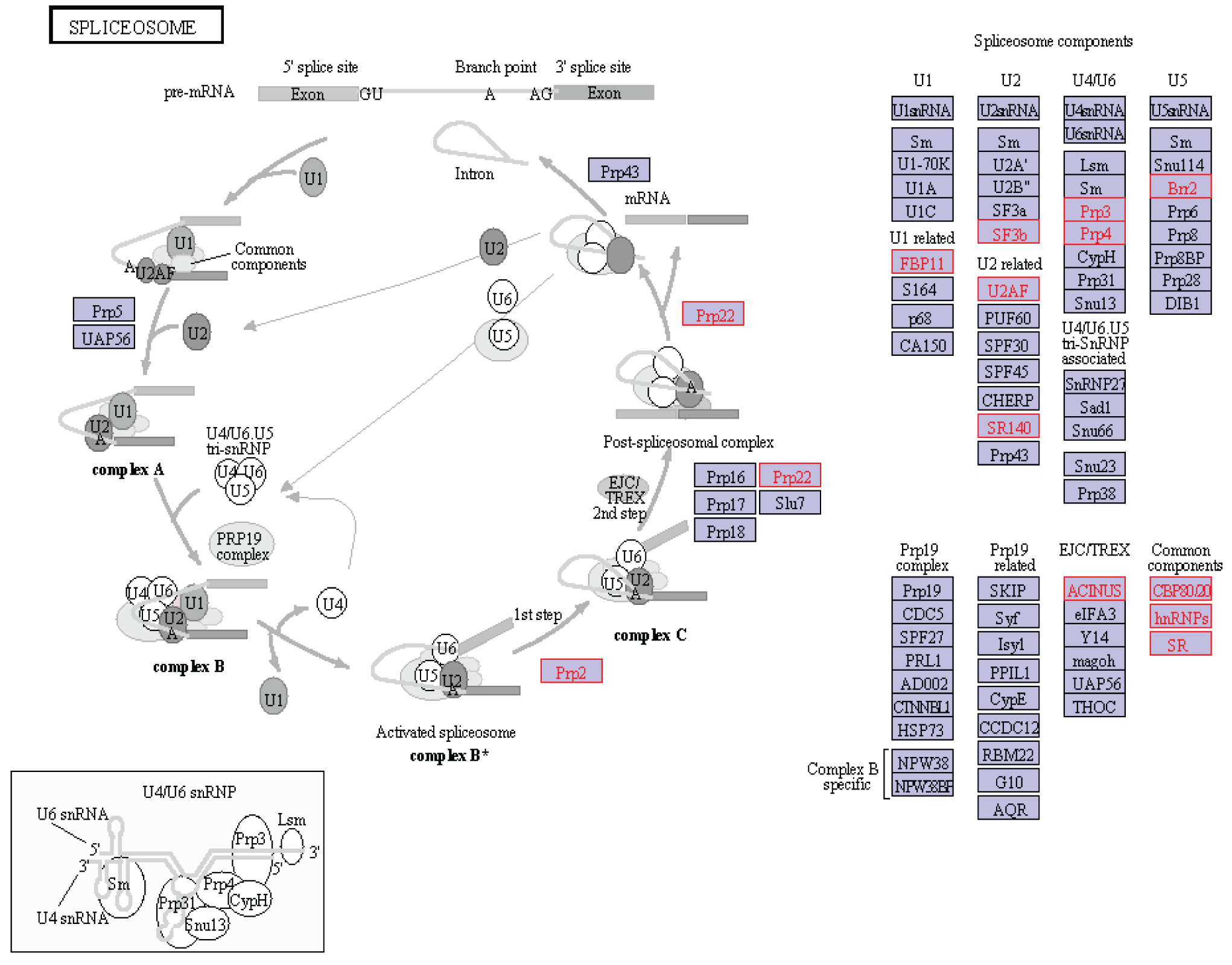

Supplement: Figure S5 — The spliceosome pathway derived from KEGG. Proteins indicated in red were found to be significantly upregulated or downregulated by SNP in our analysis. (TIF) [file pone.0094261.s005.tif]

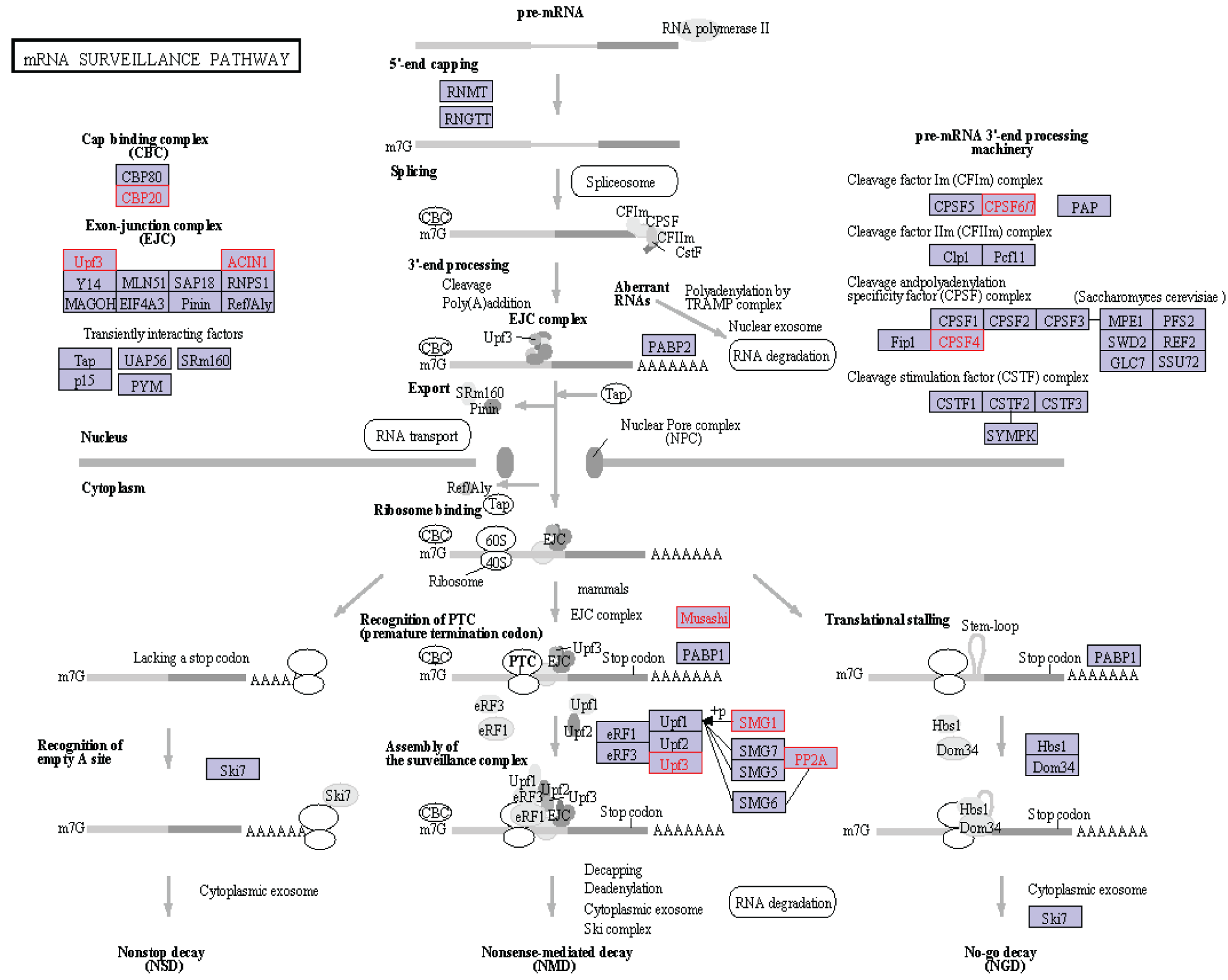

Supplement: Figure S6 — The mRNA surveillance pathway derived from KEGG. Proteins indicated red were found to be significantly upregulated or downregulated by SNP in our analysis. (TIF) [file pone.0094261.s006.tif]

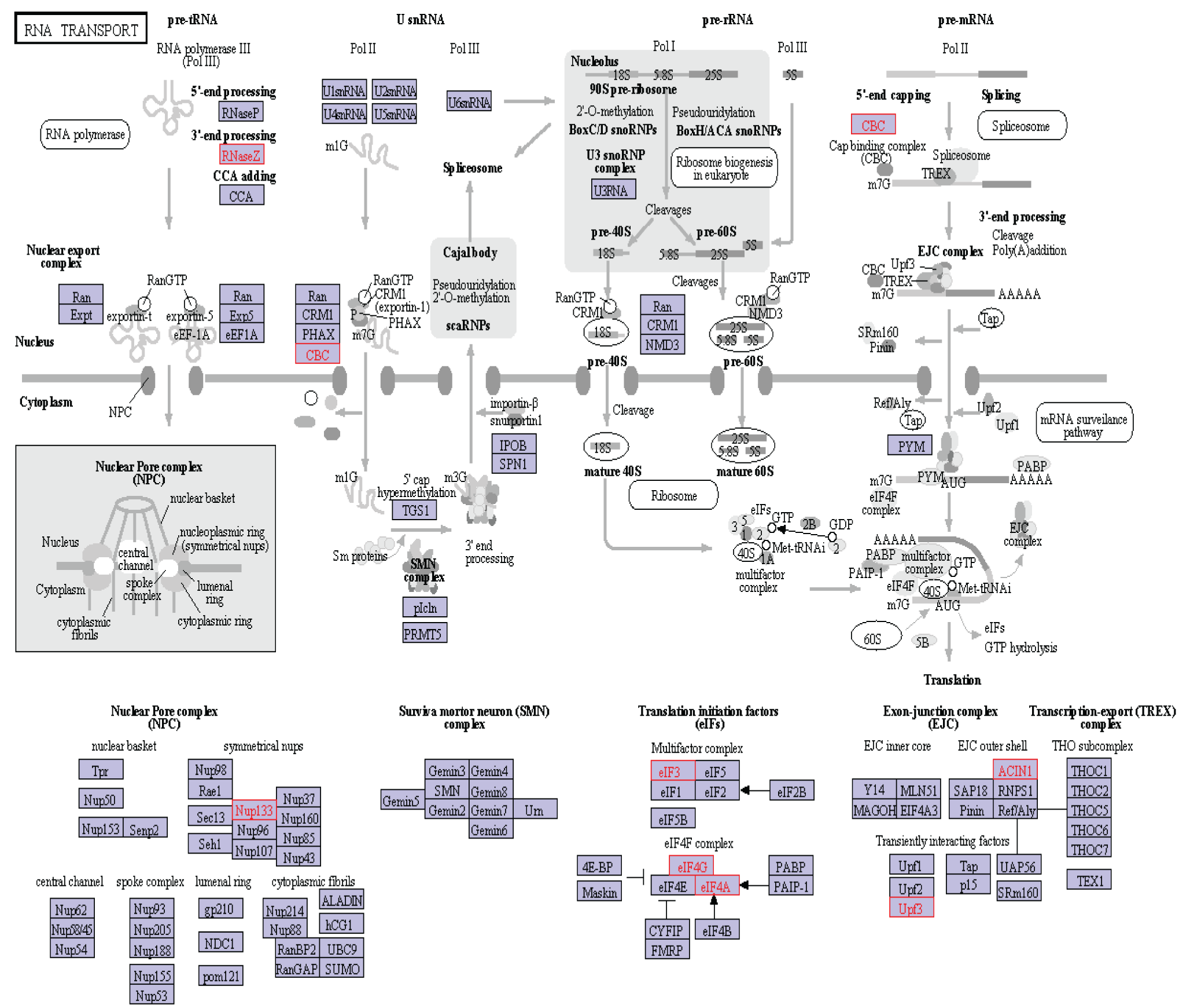

Supplement: Figure S7 — The RNA transport pathway derived from KEGG. Proteins indicated red were found to be significantly upregulated or downregulated by SNP in our analysis. (TIF) [file pone.0094261.s007.tif]

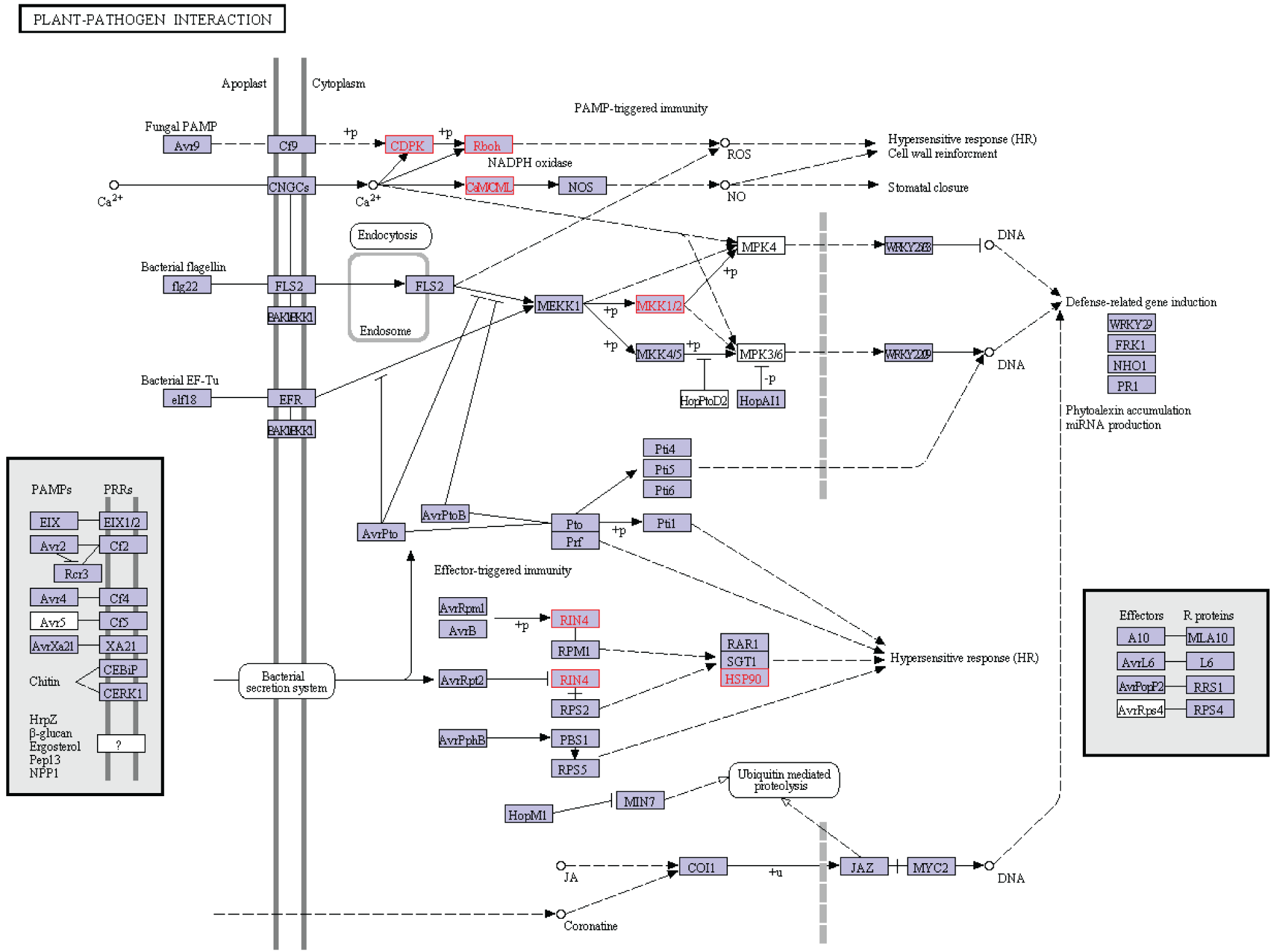

Supplement: Figure S8 — The plant-pathogen interaction pathway derived from KEGG. Proteins indicated red were found to be significantly upregulated or downregulated by SNP in our analysis. (TIF) [file pone.0094261.s008.tif]

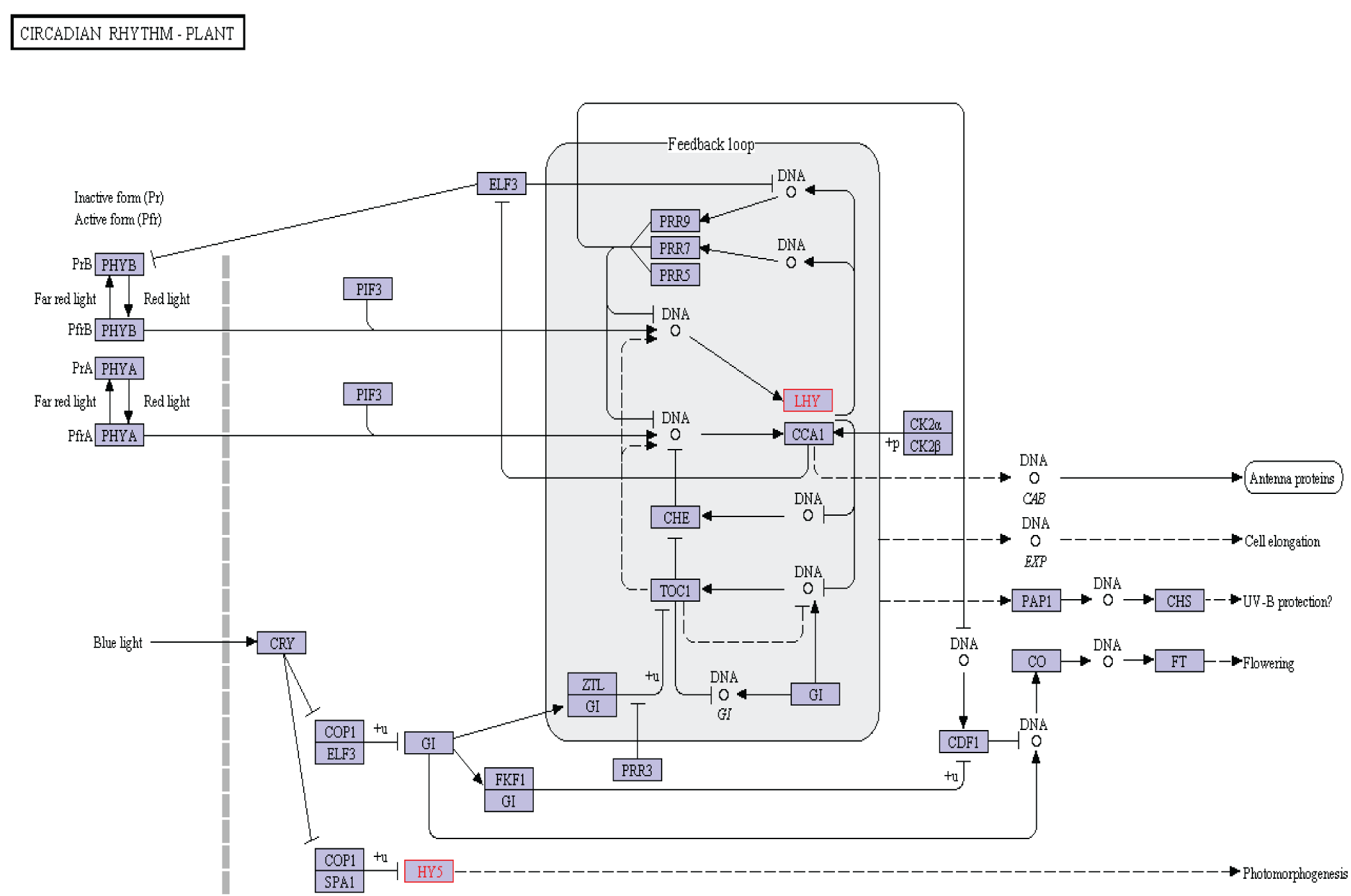

Supplement: Figure S9 — The circadian rhythm pathway derived from KEGG. Proteins indicated red were found to be significantly upregulated or downregulated by SNP in our analysis. (TIF) [file pone.0094261.s009.tif]
